# Ovariotomy

**Published:** 1869-08

**Authors:** 


### Ovariotomy.

Spencer Wells has operated 300 times. Of his first 100 cases, 34 died and 66 recovered; of the second 100, 28 died and 72 recovered; but of the third series of cases, only 23 died and as many as 77 recovered. The general mortality of the 300 cases was about 28 per cent. In those who had not been tapped, 135 in number, the mortality was about 27 per cent.; of those who had been tapped once, viz. 78 in number, the mortality was 25 per cent.; of the 36 who had been tapped twice, the mortality was 33 per cent.; or exactly the same as in those who had been tapped from 4 to 16 times. Hence one or many tappings did not materially increase the mortality of ovariotomy.

Simple cysts were sometimes cured by mere tapping, and that almost quite as often as when Iodine was also injected. Iodine injections failed when the cyst was multilocular and were only useful in deadening the fetid discharge from suppurating cysts. With this exception, the use of Iodine may be omitted, and perhaps Carbolic Acid is better.—*Medical Gazette.*

